# Multimodal reprogramming of the tumor microenvironment by MMR and dual checkpoint blockade in hepatocellular carcinoma models

**DOI:** 10.3389/fimmu.2025.1679665

**Published:** 2025-10-09

**Authors:** Mulu Z. Tesfay, Aleksandra Cios, Khandoker Usran Ferdous, Randal S. Shelton, Bahaa Mustafa, Camila C. Simoes, Murat Gokden, Isabelle R. Miousse, Kimberly J. Krager, Marjan Boerma, Alicja Urbaniak, Anuradha Kunthur, Sri Obulareddy, Joshua M. Eichhorn, Steven R. Post, Jean Christopher Chamcheu, Omeed Moaven, Chiswili Y. Chabu, Dan G. Duda, Matteo Conti, Bruno Nardo, Rang Govindarajan, Martin E. Fernandez-Zapico, Lewis R. Roberts, Mitesh J. Borad, Martin J. Cannon, Alexei G. Basnakian, Bolni M. Nagalo

**Affiliations:** ^1^ Department of Pathology, University of Arkansas for Medical Sciences, Little Rock, AR, United States; ^2^ Wintrop P. Rockefeller Cancer Institute, University of Arkansas for Medical Sciences, Little Rock, AR, United States; ^3^ Department of Pharmaceutical Sciences, Division of Radiation Health, University of Arkansas for Medical Sciences, Little Rock, AR, United States; ^4^ Department of Biochemistry and Molecular Biology, University of Arkansas for Medical Sciences, Little Rock, AR, United States; ^5^ Department of Hematology and Oncology, University of Arkansas for Medical Sciences, Little Rock, AR, United States; ^6^ College of Medicine, Radiology, University of Arkansas for Medical Sciences, Little Rock, AR, United States; ^7^ Department of Pathological Sciences, School of Veterinary Medicine, Louisiana State University, Baton Rouge, LA, United States; ^8^ Department of Biological Sciences and Chemistry, College of Sciences and Engineering, Southern University and A&M College, Baton Rouge, LA, United States; ^9^ Division of Surgical Oncology, Department of Surgery, Louisiana State University, Health Science Center, New Orleans, LA, United States; ^10^ Division of Biological Sciences, University of Missouri, Columbia, MO, United States; ^11^ Steele Laboratories for Tumor Biology, Department of Radiation Oncology, Massachusetts General Hospital and Harvard Medical School, Boston, MA, United States; ^12^ Public Health Department, Azienda Unita’ Sanitaria Locale Imola, Imola, Italy; ^13^ Department of Pharmacy, Health and Nutritional Sciences, University of Calabria, Rende, Italy; ^14^ General Surgery Unit, Department of Surgery, Azienda Ospedaliera Annunziata, Cosenza, Italy; ^15^ Schulze Center for Novel Therapeutics, Division of Oncology Research, Mayo Clinic, Rochester, MN, United States; ^16^ Department of Gastroenterology and Hepatology, Mayo Clinic, Rochester, MN, United States; ^17^ Department of Hematology and Medical Oncology, Mayo Clinic, Phoenix, AZ, United States; ^18^ Department of Microbiology and Immunology, University of Arkansas for Medical Sciences, Little Rock, AR, United States; ^19^ Department of Pharmacology and Toxicology, University of Arkansas for Medical Sciences, Little Rock, AR, United States; ^20^ Central Arkansas Veterans Healthcare System, Little Rock, AR, United States

**Keywords:** MMR vaccine, hepatocellular carcinoma, tumor microenvironment, immune checkpoint blockade, innate and adaptive immunity modulation

## Abstract

Hepatocellular carcinoma (HCC) is a leading cause of cancer-related death worldwide, thus, there is an urgent need to develop more effective therapeutic options for this dismal condition. Tumor-infiltrating lymphocytes (TILs) are associated with improved response to immune checkpoint blockade in HCC, but their low abundance in most cases limits their therapeutic efficacy. Here, we demonstrate, in mice, that low-dose intratumoral immunovirotherapy with the trivalent measles, mumps, and rubella vaccine (MMR) induces superior tumor-growth delay and extended host survival compared to individually administered vaccines for measles, mumps, or rubella viruses. Further, our results show that MMR therapy synergizes with PD-1 and CTLA-4 blockade to reprogram the tumor microenvironment, resulting in increased CD8+ TIL infiltration and reduced PD-1 expression on TILs, among other effects. These changes in the immunological landscape translated into greater survival and more durable tumor-specific and memory immune responses for hosts. Comprehensive toxicology analysis revealed no evidence of MMR-induced liver or kidney toxicity after intrahepatic administration. This work reinforces an unrecognized role of MMR plus ICB in reprogramming the immune landscape in HCC through multimodal immune activation, providing a strong rationale for further development of MMR-based therapies for HCC.

## Introduction

Hepatocellular carcinoma (HCC) is the most common form of primary liver cancer and a leading cause of cancer-related death worldwide (1). In the US, its incidence is rising faster than any other cancer, accounting for more than 30,000 deaths annually, with nearly 800,000 deaths globally (2–4). This increasing burden is largely attributed to metabolic risk factors—HCC prevalence has tripled over the past 3 decades due to rising cases of nonalcoholic fatty liver disease, obesity, and type 2 diabetes (5, 6). Although systemic therapies have improved survival outcomes, durable clinical responses remain elusive. First-line combination therapies with immune checkpoint blockade (ICB), via anti-programmed death ligand 1 (PD-L1), together with anti-VEGF and antibodies for cytotoxic T-lymphocyte-associated protein 4 (CTLA-4) have provided survival benefits in a subset of patients (5, 7–11), but response rates remain confined to less than 30% of cases, and median survival for patients with unresectable advanced HCC continues to be less than 2 years (5, 7–15).

A defining feature of ICB efficacy is the extent of tumor-infiltrating lymphocytes (TILs) in the tumor microenvironment (TME), which correlates with therapeutic outcomes (16, 17). However, most HCC tumors have a scarcity of TILs, which severely limits the immunotherapeutic potential of ICB (18, 19). Furthermore, the TME in HCC is characterized by abnormal angiogenesis, chronic inflammation, and extracellular matrix remodeling, and these features sustain an immunosuppressive niche that fosters tumor progression, invasion, and metastasis (18, 20, 21). Immunosuppressive mechanisms within the TME are major obstacles to immune surveillance, necessitating therapeutic approaches that promote TIL infiltration while also targeting pathways that promote immune escape (3, 22).

Strategies to overcome these immune barriers and immune evasion in HCC, therefore, are actively being pursued. Preclinical and clinical studies have demonstrated the efficacy of ICB with VEGF blockade in HCC (23, 24), paving the way to the US Food and Drug Administration’s approval of combination regimens with ICB that targets PD-L1 and VEGF signaling to remodel the tumor vasculature and enhance immune infiltration. Even with these advances, however, only a fraction of HCC patients derives long-term benefits from ICB, largely due to the immunosuppressive nature of the TME and the paucity of TILs. This reinforces the urgent need for novel immunotherapeutic strategies in HCC to increase immune infiltration and reprogram the TME to improve responses to ICB.

Immunovirotherapy has emerged as a promising approach to circumvent immune exclusion and enhance antitumor immunity (25–29). Among immunovirotherapies, the live trivalent measles, mumps and rubella vaccine (MMR) not only has well-established protective benefits against its targeted infectious diseases but also has potential in oncology, particularly due to its ability to reprogram the TME, which remains largely unexplored (30–32). In a preclinical HCC model, we previously demonstrated that MMR immunovirotherapy elicits antitumor immunity by augmenting cytotoxic T lymphocyte (CTL) infiltration and extends survival (26). Furthermore, MMR has demonstrated efficacy in a mouse model of colorectal cancer, prolonging survival (26), which suggests that the vaccine may have broader immunotherapeutic potential beyond HCC. While these findings highlight MMR’s therapeutic promise, it remains unclear whether MMR-driven immunovirotherapy can synergize with ICB to induce durable tumor control and extend survival in preclinical HCC models. Addressing this question is essential to establishing MMR as a novel immunotherapeutic adjuvant capable of enhancing antitumor immunity and improving clinical outcomes.

Here, we report that intratumoral MMR therapy in a mouse model of HCC not only suppressed tumor growth and extended survival but also synergized with anti-PD-1 and anti-CTLA-4 blockade to remodel the protumorigenic TME. This combination therapy enhanced CTL infiltration, reduced T-cell exhaustion, and reprogrammed immunosuppressive myeloid compartments, resulting in durable tumor-specific immunity memory in subcutaneous and orthotopic models. Importantly, we observed no clinically significant liver or renal toxicity after intrahepatic MMR administration in non-tumor-bearing mice.

These findings reveal MMR’s unrecognized immunomodulatory role in enhancing ICB therapeutic efficacy by reprogramming the HCC immune microenvironment to promote multimodal immune activation. Ultimately, this promotes durable tumor control with a favorable safety profile. This study provides preclinical evidence supporting further investigation into MMR as a potential and widely accessible adjuvant to enhance ICB efficacy, with broader implications for cancer immunotherapy.

## Results

### Evaluation of virus-induced toxicity after direct hepatic administration of MMR

**Figure 1:**
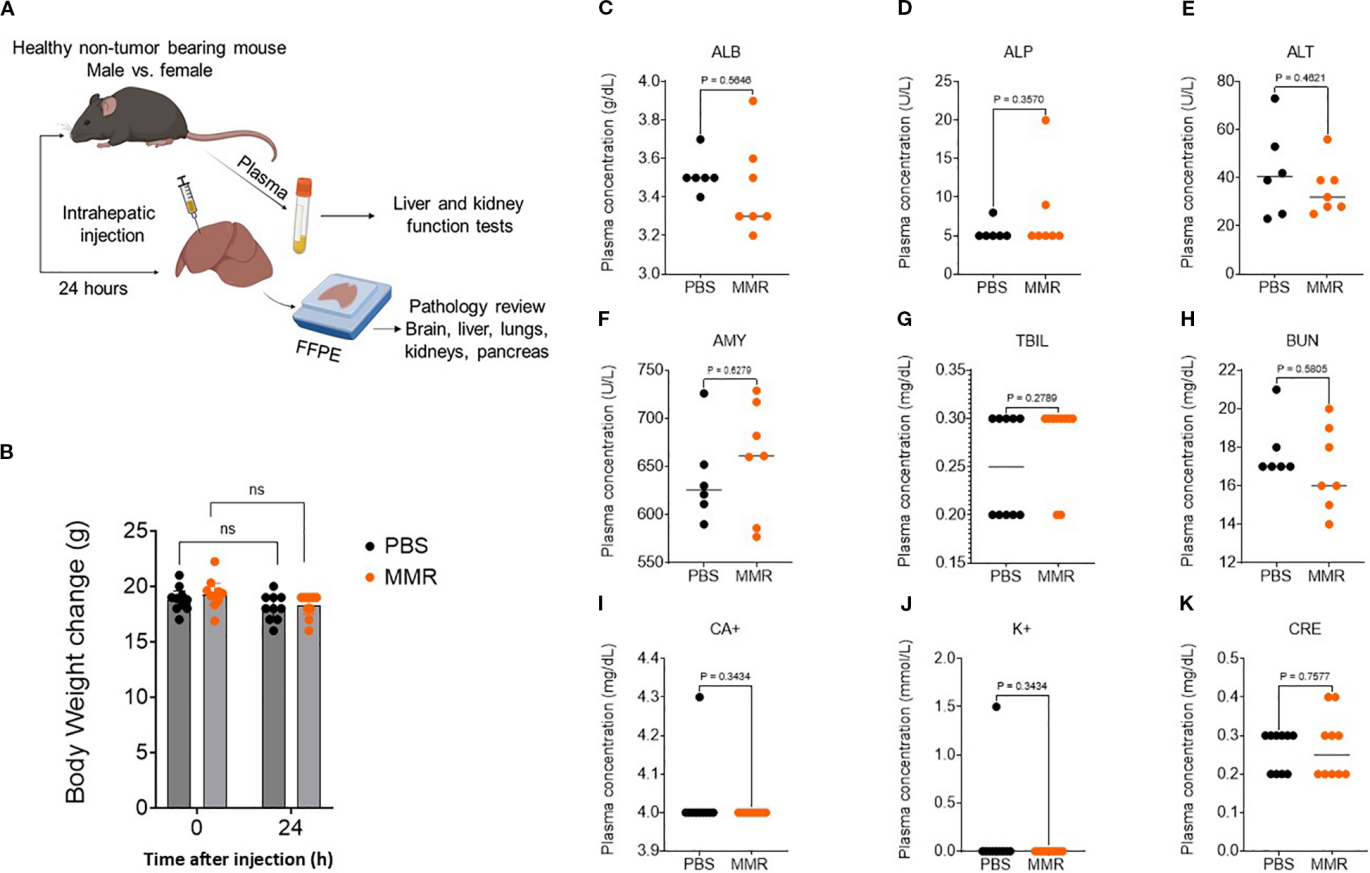
Trivalent measles, mumps, and rubella vaccine (MMR) shows no systemic toxicity. **(A)** To assess MMR-related toxicities, naïve non-tumor-bearing mice were treated with an intrahepatic injection of MMR (1 × 10^2^ TCID_50_) or PBS. Plasma was collected at 24 h to assess markers of liver toxicity, nephrotoxicity, and electrolytes. **(B)** At 24 h post-treatment, body weights were not altered by intrahepatic MMR. Plasma markers of organ toxicity—albumin (**C**, ALB), alkaline phosphatase (**D**, ALP), alanine aminotransferase (**E**, ALT), amylase (**F**, AMY), total bilirubin (**G**, TBIL), blood urea nitrogen (**H**, BUN), calcium (**I**, CA+), Potassium (**J**, K+), and creatinine (**K**, CRE)—were not altered by intrahepatic MMR.

**Figure 2:**
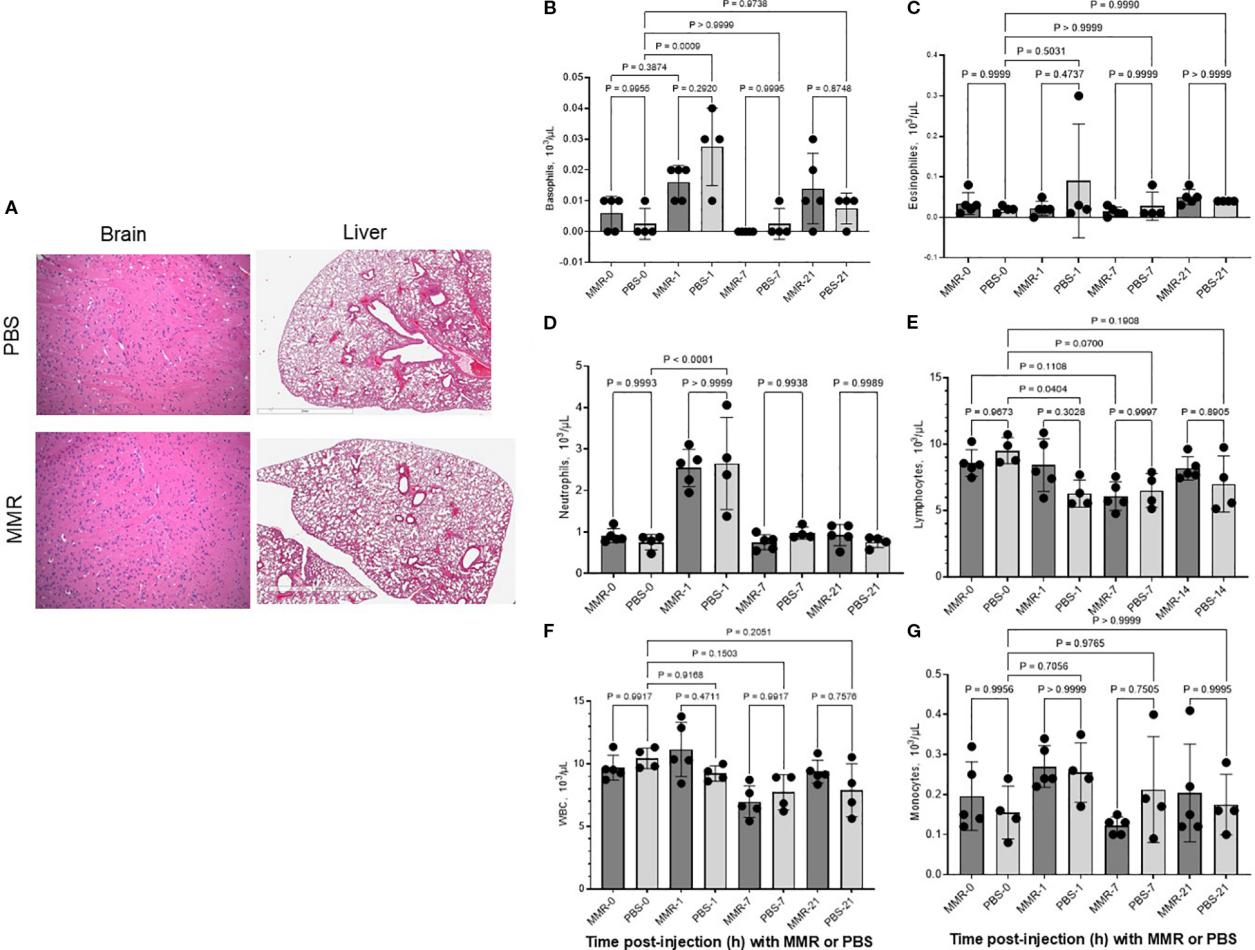
Trivalent measles, mumps, and rubella vaccine (MMR) did not affect blood cell counts and did not damage tissues. To assess MMR-related toxicities, naïve non-tumor-bearing mice were treated with an intrahepatic injection of MMR (1 × 10^2^ TCID_50_) or PBS. Blood cell counts were obtained at baseline (i.e., before intrahepatic injection; day 0) and on days 1, 7, and 21. Brain and liver tissues were harvested at the end of the experiment (21 days post-injection) and were stained and prepared for pathology analysis. **(A-G)**.

To support our MMR clinical trials underway at UAMS, we assessed potential MMR treatment-related adverse effects. C57BL/6J mice (N = 10 per group) were administered PBS or MMR at a dose of 1 × 10² TCID_50_ directly into the liver (50 µL/mouse). Body weight, body temperature, behavior, and clinical signs were monitored daily for 72 h post-injection and 3 times per week by a board-certified veterinarian to detect any signs of toxicity. To evaluate short-term toxicity, blood and tissues (brain, liver, and spleen) were harvested from 6−8 mice/group at baseline and 24 h post-infection and were subjected to hematoxylin and eosin staining (**Figure 1A**). Assessments of body weight and plasma biomarkers associated with viral-induced liver and kidney toxicity showed no evidence of significant systemic toxicity (**Figures 1B-K**) (26). Additionally, pathological analysis of brain and liver tissues revealed no treatment-related toxicities (**Figure 2A**), consistent with our previously published findings (26). Furthermore, a complete blood count (CBC) was performed at baseline (before intrahepatic injection), 24 h post-injection, and on days 7 and 21. No significant changes in blood cell composition were observed, suggesting an absence of hematologic toxicity (**Figures 2B-G**, **Supplementary Figure 1**).

### Intratumoral administration of MMR induces superior antitumor activity and survival benefits compared to individual viral components

**Figure 3:**
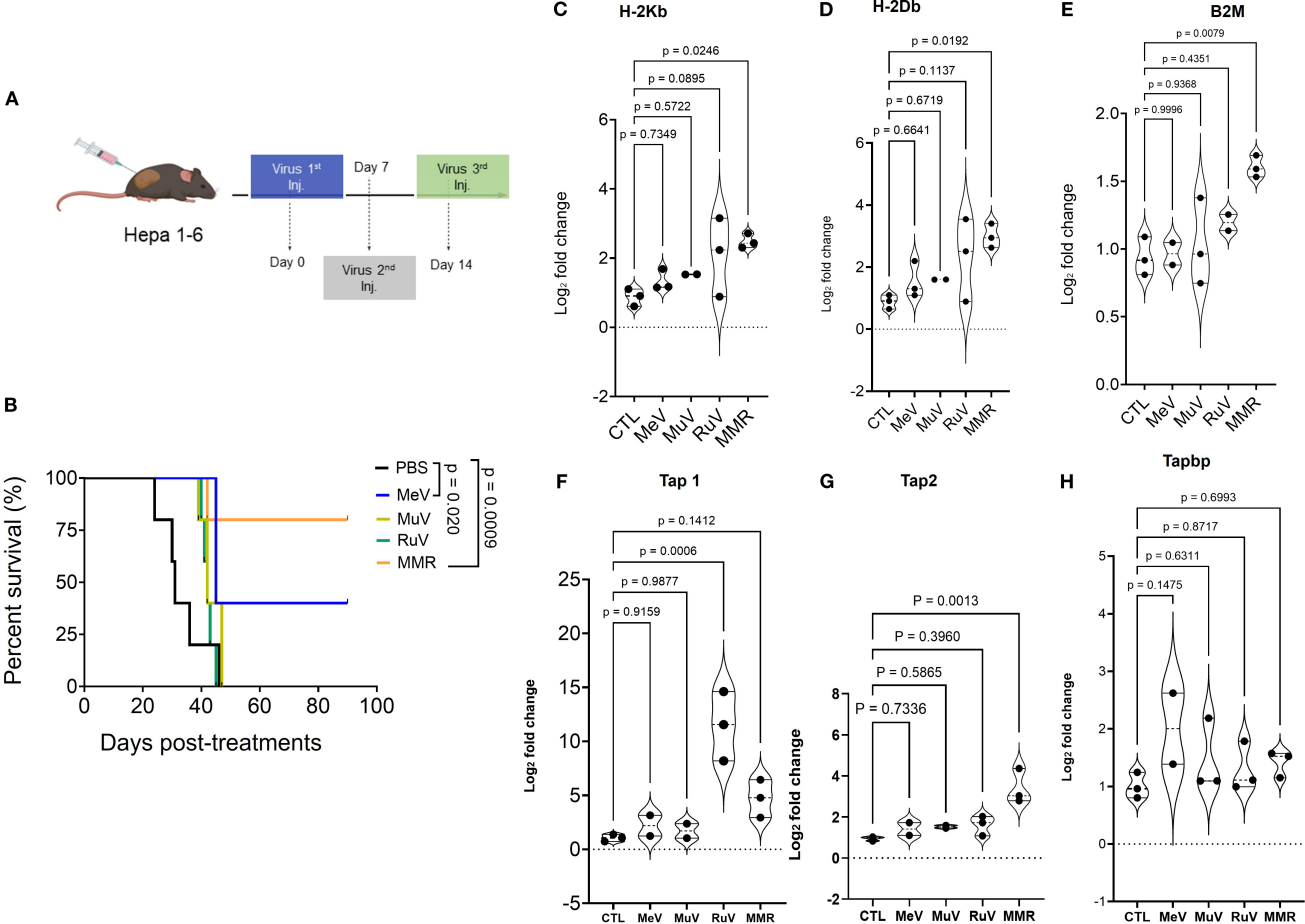
Anti-tumor activity of vaccines for individual measles (MeV), mumps (MuV), and rubella (RuV) viruses and of the trivalent vaccine (MMR) in murine HCC Hepa 1–6 model. **(A)** Hepa 1–6 tumors treated with 3 intratumoral doses (1 weekly dose for 3 weeks) of MeV, MuV, RuV, MMR (1 × 10^2^ TCID_50_ per mouse), or PBS (n = 7/group). **(B)** Survival (shown as Kaplan-Meier curves) was monitored. **(C-H)** Hepa1–6 cells grown in culture were treated with MMR or individual viruses (MeV, MuV, or RuV) for 48 h before RNA extraction and qPCR amplification to characterize MHC class I and β-2-microglobulin (B2M) expression. **(C, D)** Expression of classical murine MHC class I (H2Kb, H2Db), **(E)** B2M, and **(F-H)** transporter associated with antigen-processing (TAP 1/2 and Tapbp) complex.

To delineate the contribution of each viral component to the overall antitumor activity of the trivalent MMR vaccine, we compared its therapeutic efficacy to that of individual vaccine strains of measles (MeV), mumps (MuV), and rubella (RuV) viruses. After the *in vitro* infectivity assay of MMR and its 3 individual components (**Supplementary Figures 2A, B**), subcutaneous (SQ) tumors were established by implanting 1 ×10^6^ Hepa 1–6 cells into the right flank of mice. When tumors reached 80 to 120 mm^3^, mice received intratumoral injections of PBS or low-dose virus (1 × 10² TCID_50_ per virus) on days 0, 7, and 14 (**Figure 3A**). MMR therapy, compared to PBS treatment, significantly prolonged survival (p < 0.0009) (**Figure 3B**). Among individual viral components, MeV exhibited the strongest antitumor activity, but its efficacy remained inferior to the trivalent MMR formulation (p = 0.020) (**Figure 3B**). To assess the impact on antigen presentation, Hepa1–6 tumor cells were grown in culture, infected with MMR or its individual viral components, and analyzed with SYBR Green quantitative PCR. Results showed significant upregulation of H-2Kb (p = 0.0248), H-2Db (p = 0.0192), B2M (p = 0.0079), and Tap2 (p = 0.0013). Notably, Tap1 was selectively upregulated by MMR and RuV, but Tapbp expression was unaffected (**Figures 3C-H**). Additionally, CD8+ T cell depletion studies show CD8+ T cells are important in MMR-induced tumor control (**Supplementary Figures 2C, D**). Furthermore, mice previously immunized with MMR exhibit similar tumor control upon intratumoral MMR administration compared to naïve controls (**Supplementary Figures 2C, D**).

### MMR in combination with anti-PD-1 and anti-CTLA-4 antibodies controls tumor growth in a murine SQ HCC model

**Figure 4:**
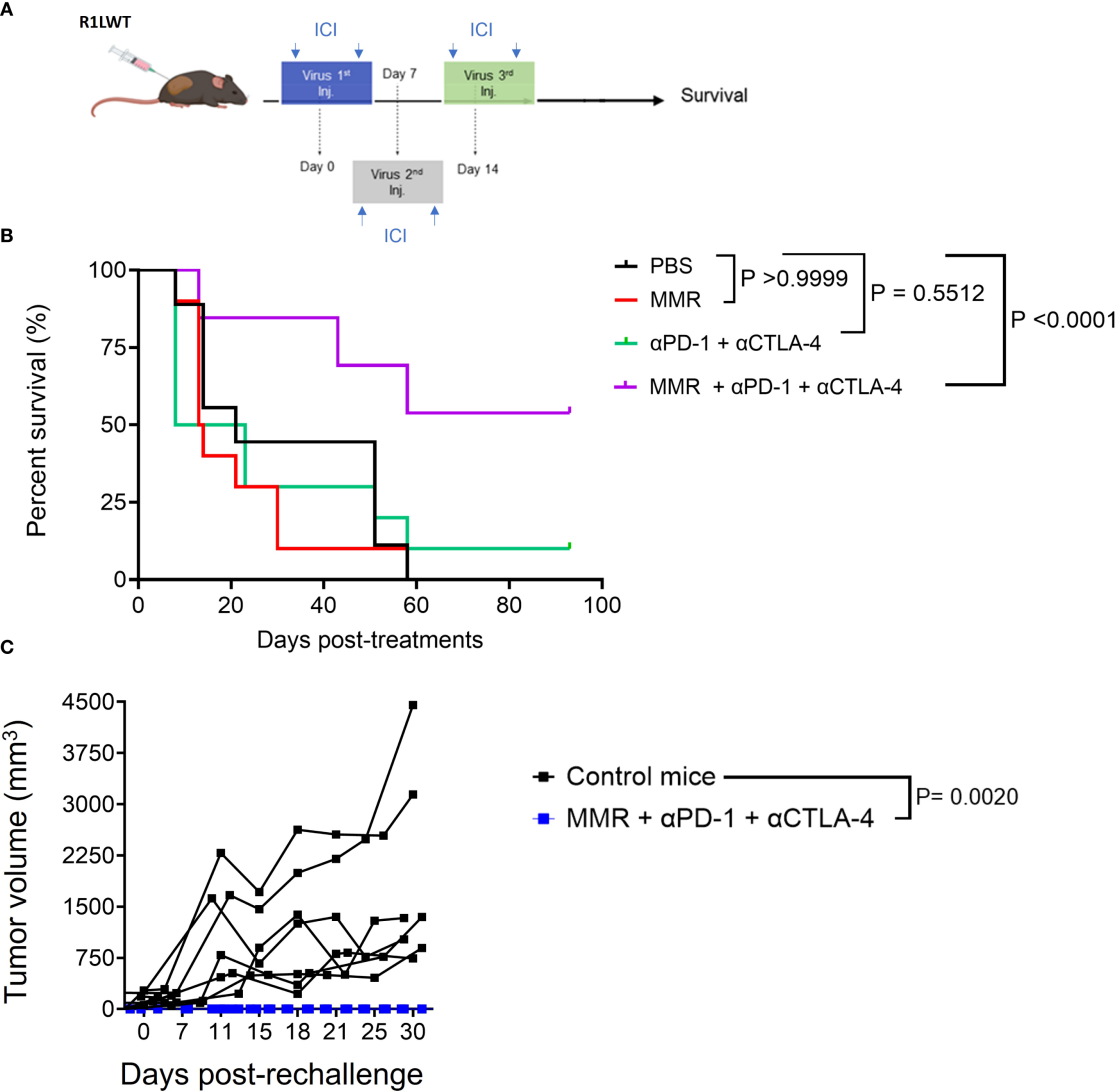
Trivalent measles, mumps, and rubella vaccine (MMR) combined with anti-PD-1 and anti-CTLA-4 prolongs survival in the murine R1LWT HCC subcutaneous (SQ) model. We investigated the anti-tumor effects of combining MMR with immune checkpoint blockade (ICB) in an immunocompetent HCC SQ mouse model (R1LWT). **(A)** We treated tumor-bearing female C57BL/6J mice with a weekly intratumoral injection of MMR (1 × 10^2^ TCID_50_) with or without ICB (intraperitoneal injections of anti-PD-1 and anti-CTLA4 antibodies; twice per week) for 3 weeks. **(B)** Survival was monitored. **(C)** shows a rechallenge result where all R1LWT-cured mice and naïve control mice were (re)challenged with SQ injection of R1LWT cells.

Next, we evaluated, in an immunocompetent SQ HCC (R1LWT, derived from RIL-175 cells) mouse model, whether ICB could potentiate the therapeutic effects of MMR, as previously described (25, 27). The R1LWT graft is an aggressive HCC murine model known to respond to blockade of PD-1 and VEGF receptor 2 (VEGFR-2) (24). Male C57Bl/6 mice bearing SQ HCC tumors were treated with intratumoral injections (once weekly for 3 weeks) of MMR (1 × 10² TCID_50_), with or without IP ICB (anti-PD-1 and anti-CTLA-4 antibodies at 5 mg/kg, twice per week for 3 weeks) (**Figure 4A**). Treatment with only MMR or ICB did not improve survival, but combination therapy with both MMR and dual-agent ICB led to significant tumor inhibition (p <0.0001), indicating a synergistic antitumor response (**Figure 4B**). To assess immunologic memory, mice that were cured of R1LWT, confirmed by the absence of palpable tumors, and treatment-naive mice were challenged with a subcutaneous injection of 5.0 × 10^5^ R1LWT cells on the left flank. Strikingly, all mice that had previously received MMR combined with dual-agent ICB completely rejected the tumor rechallenge (p = 0.0020), but treatment-naive mice developed tumors (**Figure 4C**).

### Combination therapy with anti-PD-1, anti-CTLA-4, and MMR remodels the tumor microenvironment in HCC

**Figure 5:**
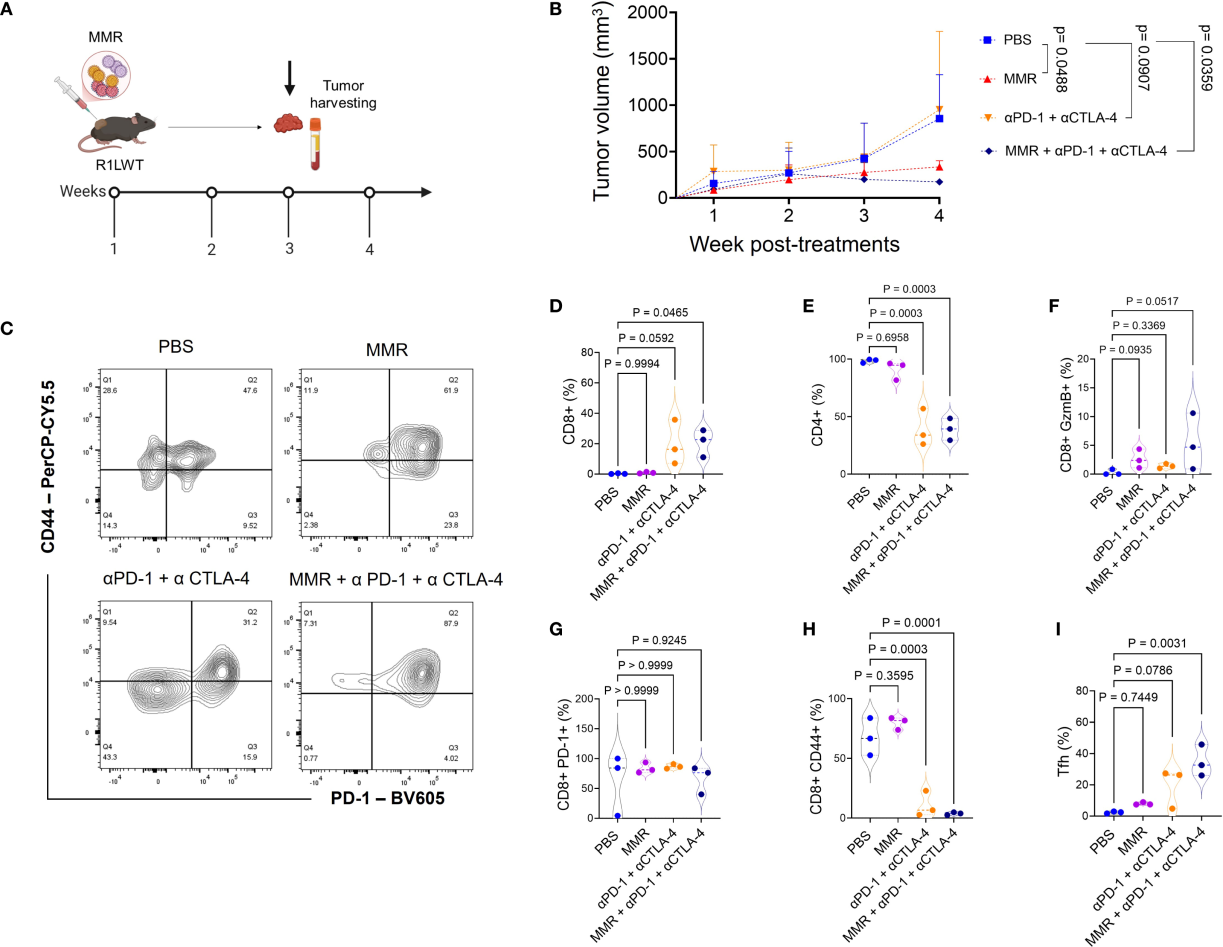
Trivalent measles, mumps, and rubella vaccine (MMR) in combination with antibodies that target PD-1 and CTLA-4 signaling enhances infiltration of cytotoxic T lymphocytes in R1LWT tumors. **(A)** Female C57BL6/J mice were implanted with R1LWT cells (n = 7/group). When the average tumor volume reached 80−120 mm^3^, PBS or MMR (1 × 10^2^ TCID_50_) was injected intratumorally on days 0, 7, and 14 with or without addition of immune checkpoint blockade (intraperitoneal injection of anti-PD-1 and anti-CTLA4 antibodies; twice per week, for 3 weeks). **(B)** Tumor volume was recorded weekly. Tumors were harvested at the end of the study for downstream analysis. **(C)** We used flow cytometry to analyze the effects on immune cell infiltration into the tumor microenvironment. **(D-I)** show levels of tumor immune infiltration (such as CD8+, PD-1+ CD44+) in ICB and the combined treatment groups.

**Figure 6:**
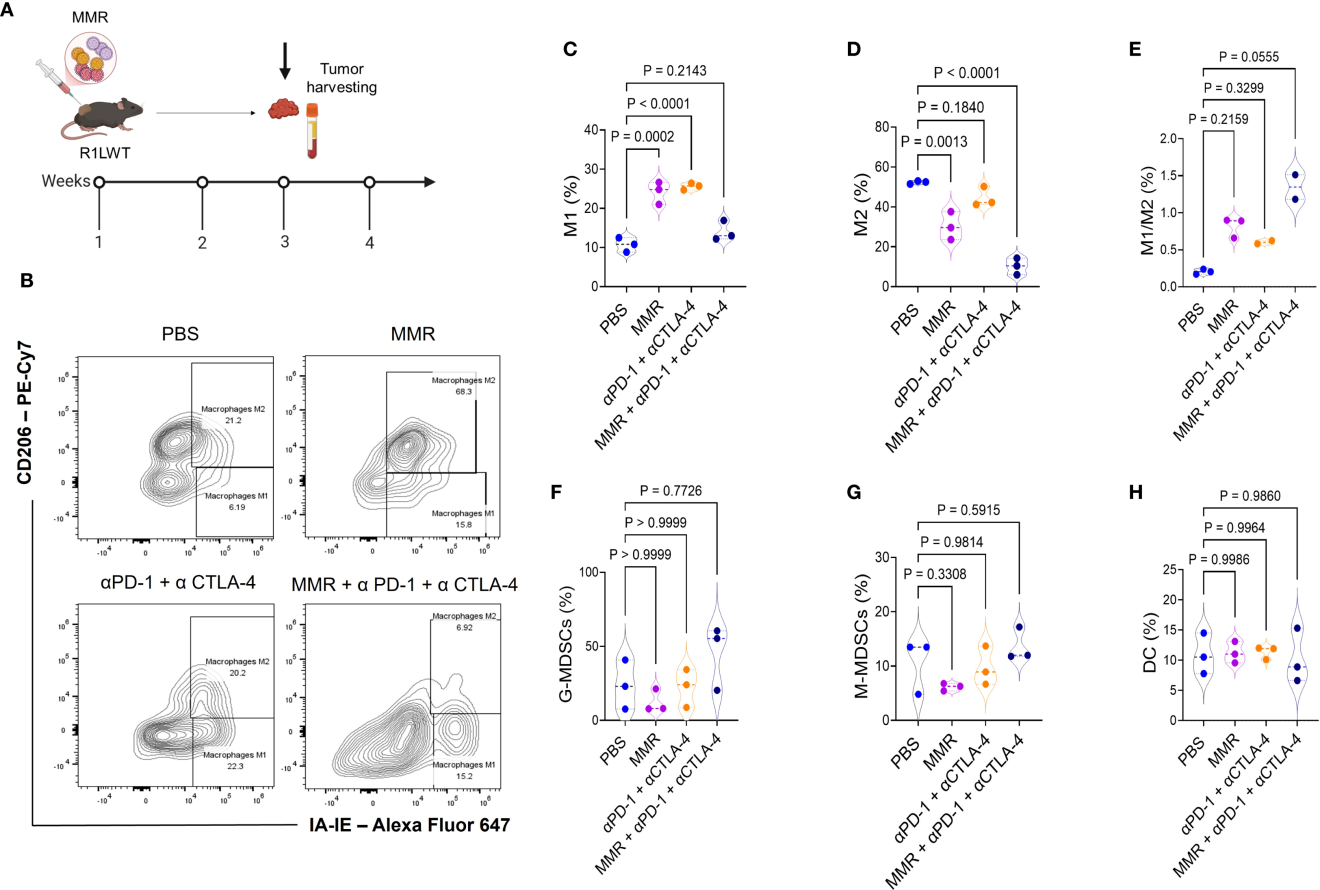
Immunotherapy treatments affect macrophage polarization differently, shifting the balance between M1 and M2 phenotypes. Female C57BL6/J mice were implanted with R1LWT cells (n = 7/group). **(A)** When the average tumor volume reached 80−120 mm^3^, mice were administered intratumoral injections of PBS or MMR (1 × 10^2^ TCID_50_) on days 0, 7, and 14 with or without addition of immune checkpoint blockade (intraperitoneal injection of anti-PD-1 and anti-CTLA4 antibodies; twice per week, for 3 weeks). **(B-H)** We harvested tumors and used flow cytometry to analyze the effects on macrophage polarization in the tumor microenvironment.

To investigate the immune mechanisms underlying the enhanced antitumor response observed with MMR and ICB combination therapy (**Figure 3**), we used flow cytometry (gating strategies shown in **Supplementary Figures 6**-**8**) to analyze immune profiles of the tumor microenvironment (TME) according to the treatment schedule outlined in **Figure 5A**. Tumors were dissociated, and immune cell populations were analyzed to determine the effects of different treatment regimens. The MMR and dual-agent ICB (i.e., PD-1 and CTLA-4 blockade) triple-combination therapy induced the most pronounced immune changes, promoting expansion of effector cells while reducing populations of immunosuppressive cells (**Figures 5C-I**). Compared to treatment with MMR-only, the combination therapy significantly increased populations of cytotoxic CD8+ T cells and double-positive CD4+ CD8+ T cells, and it expanded T follicular helper cells (**Figures 5D-I**, **Supplementary Figure 3**). Dual-agent ICB alone produced intermediate effects. Further, the triple-combination therapy reduced populations of exhausted T cells, suggesting that it may be the most effective strategy for reversing immune dysfunction (**Figure 5D**). Because tumor-associated macrophages (TAMs) contribute to immune suppression, we assessed macrophage polarization across treatment groups. Treatment with only MMR or only dual-agent ICB increased proinflammatory M1 TAMs, but MMR and the triple combination decreased immunosuppressive M2 TAMs compared to PBS (**Figures 6A-D**). As a result, the M1 to M2 ratio showed a non-significant trend toward an increase in the triple-combination group (**Figure 6E**). The changes in monocytic and granulocytic myeloid-derived suppressor cells, dendritic cells, and NK cells were not significant (**Figures 6F-H**; **Supplementary Figures 4**, **5**).

### Systemic administration of MMR with blockade of PD-1 and CTLA-4 improves survival in an orthotopic HCC model

**Figure 7:**
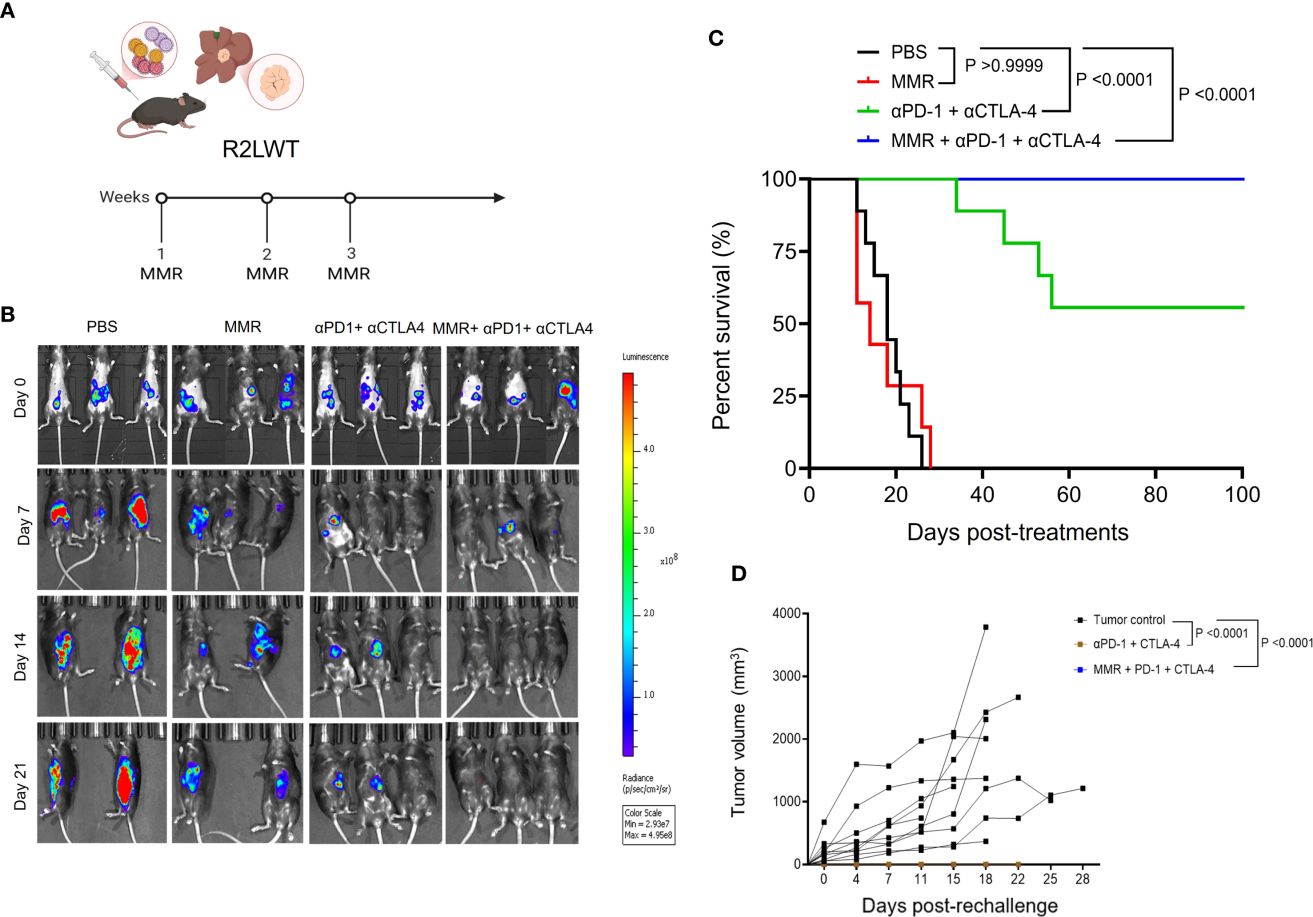
Systemic administration of MMR in combination with anti-PD-1 and anti-CTLA-4 resulted in enhanced survival in a metastatic murine R2LWT HCC model. **(A)** A metastatic HCC orthotopic mouse model was established with 5 × 10^5^ luciferase-expressing R2LWT cells surgically implanted into the livers of female C57BL/6J mice (n = 7/group). When tumors reached 4−5 mm in diameter (approximately 7 days post-implantation), mice were randomly assigned to different study groups (PBS, MMR, αPD-1+αCTLA-4 antibodies, and MMR+αPD-1+αCTLA-4 antibodies) based on IVIS imaging. For 3 consecutive weeks (on days 0, 7, and 14) mice were intraperitoneally injected with PBS or MMR (1 × 10^2^ TCID_50_). **(B)** IVIS imaging shows tumor burden. **(C)** Survival was monitored. **(D)** A subset of cured mice was rechallenged with R2LWT injected subcutaneously and compared with naïve control mice that were challenged with R2LWT; tumor volumes were monitored.

To further evaluate the therapeutic potential of MMR in combination with dual-agent ICB, bioluminescent R2LWT cells (derived from RIL-175 cells) were surgically implanted into the livers of immune-competent C57Bl/6 mice. When tumors reached 4 to 5 mm³ in volume (approximately 7 days post-implantation), which was confirmed by bioluminescence imaging, mice were randomized into treatment groups (**Figures 7A, B**). Each mouse received IP injections of either PBS, MMR (1 × 10² TCID_50_, once per week for 3 weeks), dual-agent ICB (anti-PD-1 and anti-CTLA-4 antibodies, 5 mg/kg, twice per week for 3 weeks), or triple-combination therapy with MMR and dual-agent ICB. The tumor burden was monitored with bioluminescence imaging before and after treatment. Our data showed that the dual-agent ICB potentiated the antitumor activity of MMR, leading to significantly reduced tumor burden and prolonged survival (**Figures 7B, C**; **Supplementary Figure 9**). To assess long-term immune protection, all surviving mice with controlled tumor growth (i.e., 2 consecutive negative bioluminescence imaging assessments) were rechallenged with SQ implantation of the same strain of HCC cells. As expected, all previously treated mice completely rejected the rechallenge, but treatment-naive mice developed tumors, indicating that the triple-combination therapy induced a durable antitumor immune response (**Figure 7D**).

## Discussion

Viral-based immunotherapies have gained increasing interest in oncology due to their ability to target tumor cells directly while simultaneously stimulating host antitumor immunity (25, 26, 34–37). Although genetically engineered measles and mumps viruses have demonstrated promise as oncolytic agents in preclinical and clinical studies (38–40), their translation into routine clinical use necessitates extensive genetic modifications and regulatory approval. In contrast, MMR presents a readily available, “off-the-shelf” immunotherapeutic platform with a well-documented safety profile and broad global accessibility (41–44).

Our findings in preclinical models provide compelling evidence that MMR reprograms the immunosuppressive TME of HCC, enhancing the efficacy of standard ICB. Building on the immunostimulatory potential of MMR (26), we demonstrated that, in the Hepa 1–6 tumor model, the antitumor activity of intratumoral MMR therapy is superior to that of its individual viral components. Additionally, *in vitro* infection of murine Hepa 1–6 cells with MMR, relative to infection with the individual components, upregulated genes essential for antigen processing and presentation (i.e., H-2Kb, H-2Db, B2M, Tap2) to a greater extent, suggesting that the three live attenuated viruses may cooperate to enhance antigen processing and presentation, ultimately improving immune recognition and antitumor efficacy. This is consistent with findings showing that transcriptomic changes in TILs correlated with increased numbers of antitumor γδ T cells in MMR-vaccinated individuals (30). Our studies further suggest that CD8+ T cells are essential contributors to the MMR-induced antitumor activity. Interestingly, previously immunized mice with MMR exhibited similar tumor control upon intratumoral MMR administration compared to naïve controls, suggesting that preexisting immunity may not significantly compromise the therapeutic efficacy of MMR-based virotherapy when administered directly into the tumor. This supports the feasibility of using MMR in previously vaccinated patient populations.

In the R1LWT SQ murine HCC model, intratumoral administration of MMR combined with antibodies that block PD-1 and CTLA-4 (i.e., triple-combination therapy) suppressed tumor growth and reprogrammed the TME by enhancing CTL infiltration, reducing PD-1+ exhausted T cells, and polarizing TAMs. Comprehensive immune profiling of tumors treated with triple-combination therapy revealed increased numbers of T follicular helper cells. These changes in immune cells were associated with improved therapeutic efficacy in the R2LWT orthotopic murine HCC model, where systemic administration of MMR combined with antibodies that block PD-1/CTLA-4 resulted in tumor control that was superior to that resulting from either treatment alone. This outcome likely indicates that ICB-induced antitumor immunity is enhanced by MMR-mediated immune system engagement.

The immunological complexity of HCC requires tailored therapeutic approaches that account for tumor-specific immune profiles (18, 20). In the SQ R1LWT HCC model, MMR combined with dual-agent ICB significantly improved survival (~55%), compared to treatment with dual-agent ICB alone (~10%), demonstrating a synergistic effect between viral-mediated immune priming and checkpoint blockade. However, in the R2LWT orthotopic model, which is more immunogenic, dual-agent ICB alone was equally effective as triple-combination therapy, suggesting that baseline tumor immunogenicity influences the need for additional viral stimulation in this model.

A major advantage of MMR over bioengineered oncolytic viruses is its established safety and regulatory approval for human use. While recombinant measles and mumps viruses require high therapeutic doses (~1 × 10^11^ TCID_50_) (39, 45, 46) and extensive clinical validation, MMR (~1 × 10^3^ TCID_50_) is widely available and is supported by decades of real-world safety data. Evaluation of systemic toxicity in non-tumor-bearing mice confirmed that intrahepatic MMR administration did not induce hepatotoxicity, nephrotoxicity, or hematologic abnormalities, which supports its safety for loco-regional use. This is particularly relevant in HCC, where loco-regional therapies such as transarterial chemoembolization, radioembolization, and cryoablation are standard practice (47–49). Integrating MMR with these interventions could enhance tumor immunogenicity and immune cell infiltration while maintaining treatment tolerability; therefore, additional investigation is warranted.

While the results are promising, our study has limitations that must be addressed before the results can be clinically translated. First, we used preclinical models of HCC that do not fully recapitulate the fibrotic and inflammatory TME of human HCC, particularly in cirrhosis, fibrosis, and metabolic-dysfunction-associated steatotic liver disease. Because most HCC cases arise in the setting of chronic liver disease, future studies should incorporate physiologically relevant models, such as the carbon tetrachloride-induced fibrosis model (50), which will facilitate assessments of MMR’s immunomodulatory effects in cirrhotic and non-cirrhotic HCC. Second, murine models lack preexisting immunity to MMR, which could significantly influence responses in human patients. However, we acknowledge that our limited study on immunized mice argues against such an effect. MMR vaccination is widespread in the US population, so preexisting memory T-cell and B-cell responses in patients with cancer may amplify vaccine-induced immune activation within the TME, potentially enhancing ICB efficacy. Evaluating the impact of MMR-specific immune recall in patients will be crucial for determining its potential to overcome resistance mechanisms.

While MMR-induced remodeling of the immune cell landscape was observed, the precise mechanisms driving tumor-specific immune responses remain to be fully elucidated. A broad immunostimulatory effect is indicated by the observed increases in CTLs, T follicular helper cells, NK cells, and proinflammatory macrophages, alongside a reduction in exhausted PD-1+ T cells. However, the relative contributions of innate versus adaptive immunity, the durability of immune memory, and the specific antigenic targets that drive MMR-induced responses require additional investigation. Lastly, the heterogeneity of HCC suggests that not all tumors will respond equally to MMR-based therapy. The variations in responses of our HCC models highlight the need for biomarker-driven stratification to identify patients most likely to benefit from MMR immunovirotherapy.

Importantly, integrating MMR-based immunovirotherapy into existing HCC surveillance and management protocols could significantly enhance patient monitoring and treatment stratification. Commonly used clinical biomarkers such as alpha-fetoprotein (AFP) and des-gamma-carboxy prothrombin (DCP) could serve as valuable tools to assess therapeutic response and guide treatment decisions in real time. In addition, leveraging emerging predictive algorithms that incorporate tumor antigenicity, immune infiltration patterns, and systemic immune profiles—as described in (51)—could enable patient stratification based on immunological responsiveness. Such precision approaches may help identify HCC patients most likely to benefit from MMR-based therapy, thereby maximizing efficacy while minimizing unnecessary treatment exposure.

This study establishes MMR as a clinically accessible, cost-effective, and immunologically potent immunovirotherapy capable of reprogramming the TME and enhancing ICB efficacy in HCC. Unlike bioengineered oncolytic viruses, which require extensive modification, MMR is a widely available, multimodal immunotherapeutic strategy that enhances antigen presentation, increases CTL infiltration, and promotes immune memory.

Future research priorities should include patient stratification, assessment of MMR-induced immune recall in preclinical models before clinical translation, and evaluation of integrating MMR with standard-of-care regimens for HCC and other solid malignancies. A more detailed understanding of MMR’s immunomodulatory mechanisms, its effects on fibrotic TME, and the durability of tumor-specific immunity will be essential for advancing clinical development.

Due to its unique immunostimulatory properties, MMR has the potential to expand access to effective cancer immunotherapy and to overcome resistance mechanisms that limit current treatments. The findings reported here support the need for further clinical investigation to evaluate MMR’s role as an immunotherapeutic adjuvant that has broader implications for enhancing antitumor immunity beyond HCC.

## Methods

### Cells and culture conditions

The murine hepatoma Hepa 1-6 (ATCC CRL-1830) cell lines used in this study were purchased from ATCC. R1LWT, R2LWT, and RIL-175 cells were obtained from Dan G. Duda, PhD (24), Massachusetts General Hospital, Boston, MA. All cells were cultured in Dulbecco’s modified eagle medium supplemented with 10% fetal bovine serum, 1% L-glutamine, and 1% penicillin/streptomycin. All cells were tested for mycoplasma and passaged in a tissue culture incubator at 37 °C and 5% CO_2_.

### Bioluminescence imaging of orthotopic HCC

Tumor growth and treatment response were monitored with a noninvasive imaging procedure using an IVIS Xenogen imaging system. Tumor-bearing mice were anesthetized with isoflurane and injected intraperitoneally (IP) with D-luciferin (ThermoFisher #88292; 50 mg/kg body weight in 100 µL PBS per mouse). Subsequently, mice were imaged once per week (days 0, 7, and 14) with an IVIS Xenogen imaging system to assess tumor growth and virus-induced changes in tumor growth, as described previously (26).

### Preparation of the trivalent live attenuated MMR

The MERCK live attenuated MMR vaccine was purchased from the University of Arkansas for Medical Sciences (UAMS) pharmacy and contained attenuated live Edmonston measles, B level Jeryl Lynn mumps, and RA 27/3 Rubella viral strains. A single immunizing dose (individual 500 μL vial) of the MMR vaccine delivers 1 × 10^3^, 1 × 10^4^, and 1 × 10^3^ median tissue culture infectious doses (TCID_50_) of attenuated measles, mumps, and rubella viruses, respectively. This study used a dose that is 10-fold lower (1 × 10^2^ TCID_50_ for measles virus and for rubella virus; 1 × 10^3^ TCID_50_ for mumps virus) than the immunizing dose. To prepare the vaccine for animal studies, lyophilized MMR vaccine powder vials were reconstituted and diluted with the provided diluents as recommended by the manufacturer (Merck). Vaccines for the individual measles (VR-24), mumps (VR-106), and rubella (VR-1359) viruses were purchased from ATCC.

### Animal studies

Female C57BL/6J mice (RRID: IMSR_JAX: 000664) and male C57BL6/J mice (RRID: IMSR_JAX: 000664) were purchased from Jackson Laboratories at 6–8 weeks of age. All mice were housed at the Division of Laboratory Animal Medicine at UAMS. The facility employs a full staff of veterinarians and veterinary technicians who supervise and assist with animal care throughout the studies. All animal procedures were performed in accordance with institutional and national guidelines for humane animal care and use. Mice were euthanized using carbon dioxide (CO_2_) inhalation, delivered at a flow rate of 30–70% of the chamber volume per minute, followed by cervical dislocation to ensure death. This method complies with the American Veterinary Medical Association (AVMA) guidelines and was approved by the University of Arkansas for Medical Sciences Institutional Animal Care and Use Committee (IACUC).

### HCC orthotopic mouse models

The orthotopic tumor model was established with 5 × 10^5^ luciferase-expressing R1LWT, R2LWT, or RIL-175 cells surgically implanted into one lobe of the liver of each C57BL/6J mouse (males and females were used in equal numbers). When tumors reached 4 to 5 mm in diameter (approximately 7 days post-implantation), mice were randomly assigned to different study groups. For 3 consecutive weeks (on days 0, 7, and 14), IP injections of PBS or MMR vaccine were administered with or without the addition of dual-agent ICB (i.e., anti-PD-1 and anti-CTLA4 antibodies, 5mg/kg; BioXCell). Tumor sizes were measured with bioluminescence imaging 14 days after tumor implantation for animal randomization and once per week for 60−90 days. Body weights were measured twice per week. During the first week of treatment and after each injection, mice were monitored daily for signs of recovery for up to 72 h. Mice were euthanized when body weight loss exceeded 20% or for tumor burden. Mortality during the survival study was assessed with the log-rank test to compare the differences in Kaplan-Meier survival curves.

### Blood chemistry and cytokines

Plasma was prepared from samples of peripheral blood that were collected via orbital bleeds 24 h after MMR vaccine administration. A blood chemistry analyzer (Abaxis Piccolo Xpress chemical analyzer) was used for blood chemistry analysis to assess markers of liver toxicity (i.e., aspartate transaminase, alkaline phosphatase, albumin), nephrotoxicity (i.e., creatinine, blood urea nitrogen), and plasma electrolytes.

### Flow cytometry antibody analysis

The antibodies used for flow cytometry analysis are presented in **Supplementary Table 1**.

### Gating strategy and subsets of tumor-infiltrating leukocytes

To obtain a single-cell suspension of tumor-infiltrating leukocytes for immune profiling, tumors were digested on a gentleMACS Dissociator (Miltenyi Biotec) with the mouse Tumor Dissociation Kit (Miltenyi Biotec), according to the manufacturer’s instructions, and then dissociated by passing the cells through a 30-μm cell strainer (Miltenyi Biotec). Cells were washed with PBS (ThermoFisher) and 1% FCS (ThermoFisher) (centrifugation at 500 g, 5 min, 25 °C) and then counted, using trypan blue stain on Invitrogen Countess 3 Automated Cell Counter (ThermoFisher). Cells then were stained with fluorochrome-conjugated antibodies in the appropriate ratio (for live/dead stain, 0.5 µl:106 cells; for all other antibodies, 1µl:106 cells). After 30 min of incubation, samples were washed 2 times with PBS (centrifugation at 500 g, 5 min, 25 °C), and cell pellets were resuspended in 100 µl of PBS, fixed, and analyzed with Cytek Northern Lights cytometer at the UAMS Flow Cytometry Core. All immune lineages were subsequently analyzed from CD45+ populations. We gated for CD45 and CD3 (T-cell marker); from the CD45+CD3+ population, we used the presence of CD4 and CD8 surface markers to identify helper T cells (CD4+CD8−), double-positive T cells (CD4+CD8+), and cytotoxic T cells (CD4−CD8+). Further, within populations of both helper and cytotoxic T cells, we gated for CD44+ and CD279+ (i.e., PD-1) cells to identify activated T cells. Within the population of cytotoxic T cells, we also identified Granzyme B+ cells.

Secondly, from the CD45+CD3− population, we gated for CD11b+ cells to further stratify the myeloid-derived suppressor cells (MDSC) population according to levels of Ly6C and Ly6G.

Thirdly, within the CD45+CD3− population, we also gated for markers to stratify hematopoietic cells. From the CDF4-80+CD11b+ subset, we stratified macrophages and their subpopulations: M1 macrophages were defined as CDIA-IE+CD206−, and M2 macrophages were defined as CDIA-IE+CD206+. Dendritic cells were stratified according to surface expression of CD11b and CD11c, and natural killer (NK) cells according to the expression of CD11b and CD335.

Results were analyzed with FlowJo software v10.10 (BD Biosciences).

### Quantitative real-time reverse transcription–polymerase chain reaction

RNA was extracted from tumors with the RNeasy kit (QIAGEN) according to the manufacturer’s instructions. The amount and quality of RNA was determined with spectrophotometry (Nanodrop). As directed by the manufacturer, reverse transcription was carried out with the iScript Reverse Transcription Supermix (Bio-Rad). The iTaq Universal SYBR Green Supermix (Bio-Rad) was used to amplify cDNA for each quantitative real-time PCR assay. The relative quantity of mRNA was determined with the delta-delta CT method, with RPLP0 serving as a housekeeping gene, as previously described (33). The following primers were used: forward (measles)- 5’CCT CAA TTA CCA CTC GAT CCA G 3’, reverse (measles)- 5’ TTA GTG CCC CTG TTA GTT TGG 3’; forward (mumps)- 5’ TCA AGC CAG AAC AAG CCT AG 3’, reverse (mumps)- 5’ TTG ATA ACA GGT CCA GGT GC 3’; and forward (rubella)- 5’ TTG AAC CTG CCT TCG GAC 3’, reverse (rubella)-5’ CCT GGT CTC TGT ATG GAA CTT G 3’.

### Statistical analysis

All values were expressed as the mean ± standard error of the mean, and the results were analyzed with one-way analysis of variance and t-test to compare group means. The Kaplan-Meier survival method was used to examine mouse survival. All tests were performed with statistical software in GraphPad Prism, version 8 (GraphPad Software). Statistical significance was defined as p <0.05.

## Data Availability

The raw data supporting the conclusions of this article will be made available by the authors, without undue reservation.

## Supplementary material

Supplementary Figure 1 Comprehensive blood cell counts in non-tumor-bearing mice treated with the trivalent measles, mumps, rubella vaccine (MMR). Naïve non-tumor-bearing mice were treated with an intrahepatic injection of MMR (1 × 10^2^ TCID_50_) or PBS control. **(A-K)** Complete blood cell counts at baseline before intrahepatic injection (day 0) and on days 1, 7 and 21 after intrahepatic injection of MMR show no significant changes, compared to time-matched controls, which received PBS.

Supplementary Figure 2 Assessment of individual virus gene expression. **(A-C)** To confirm *in vitro* infectivity, murine HCC cells (R1LWT) were infected with the trivalent measles, mumps, and rubella vaccine (MMR) or with the individual viruses (MeV, MuV, RuV) for 48 h before RNA extraction and qPCR amplification of viral RNA. **(D, E)** MMR immunization improves anti-tumor activity, and *in vivo* CD8 depletion decreases MMR anti-neoplastic effect. PBS (Vehicle) represents MMR immunization followed by IT PBS injection, PBSim represents PBS immunization followed by MMR IT injections, MMRim represents MMR immunization followed by MMR IT injections, MMRim/αIgG represents MMR immunization followed by αIgG antibody treatment and MMR IT injections, MMRim/αCD8 represents MMR immunization followed by αCD8 antibody treatment and MMR IT injections, as depicted in **(D)**.
