# Supplementary material for: Multimodal reprogramming of the tumor microenvironment by MMR and dual checkpoint blockade in hepatocellular carcinoma models
Source: Front Immunol. 2025 Oct 9;16:1679665. doi: 10.3389/fimmu.2025.1679665 (PMC12546120; doi:10.3389/fimmu.2025.1679665)
Supplement: Supplementary Figure 1 — Comprehensive blood cell counts in non-tumor-bearing mice treated with the trivalent measles, mumps, rubella vaccine (MMR). Naïve non-tumor-bearing mice were treated with an intrahepatic injection of MMR (1 × 10^2^ TCID50) or PBS control. (A-K) Complete blood cell counts at baseline before intrahepatic injection (day 0) and on days 1, 7 and 21 after intrahepatic injection of MMR show no significant changes, compared to time-matched controls, which received PBS. [file DataSheet1.pdf]

Supplementary data

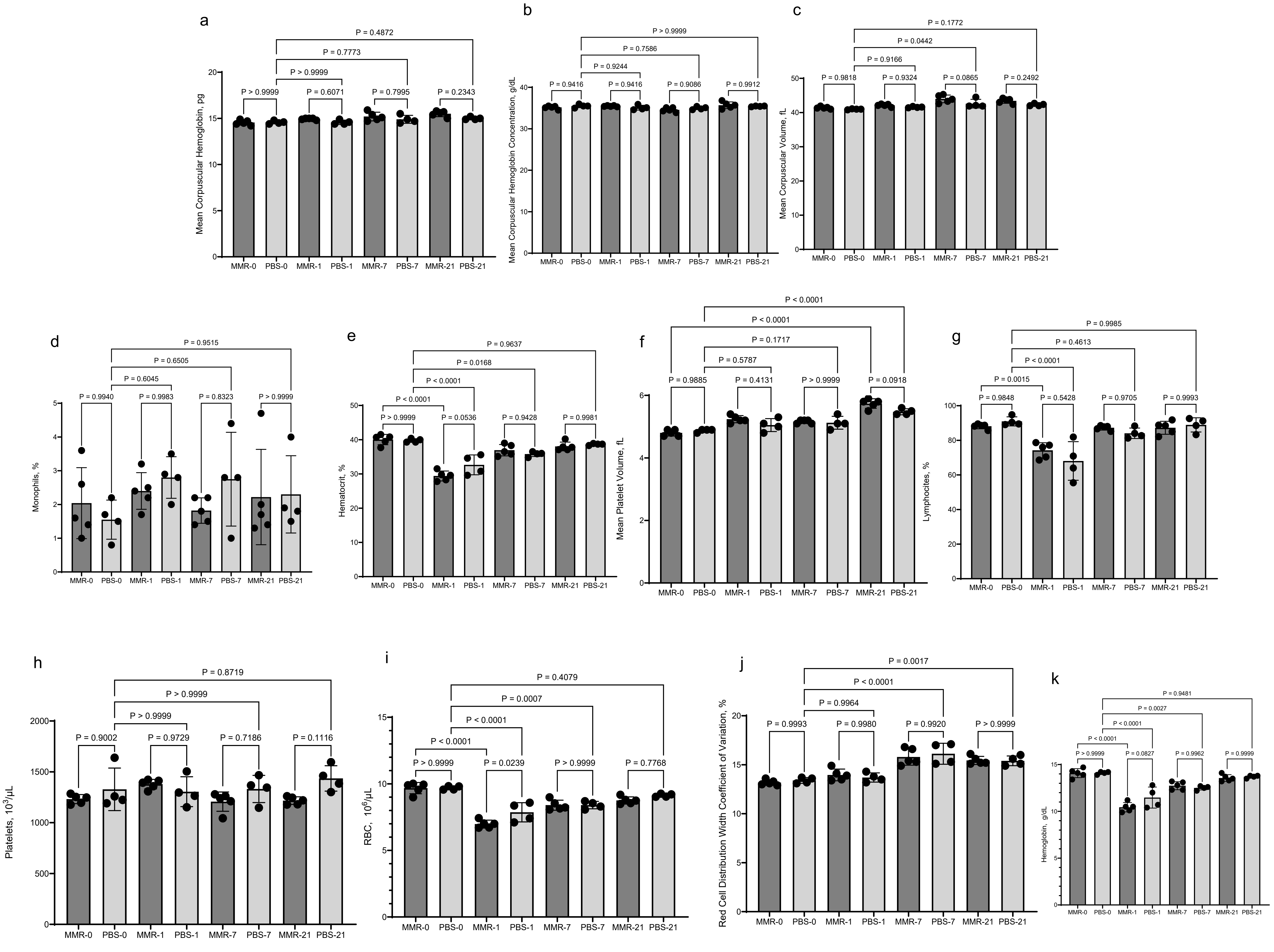

Supplementary Figure 1. Comprehensive blood cell counts in non-tumor-bearing mice treated with the trivalent measles, mumps, rubella vaccine (MMR).

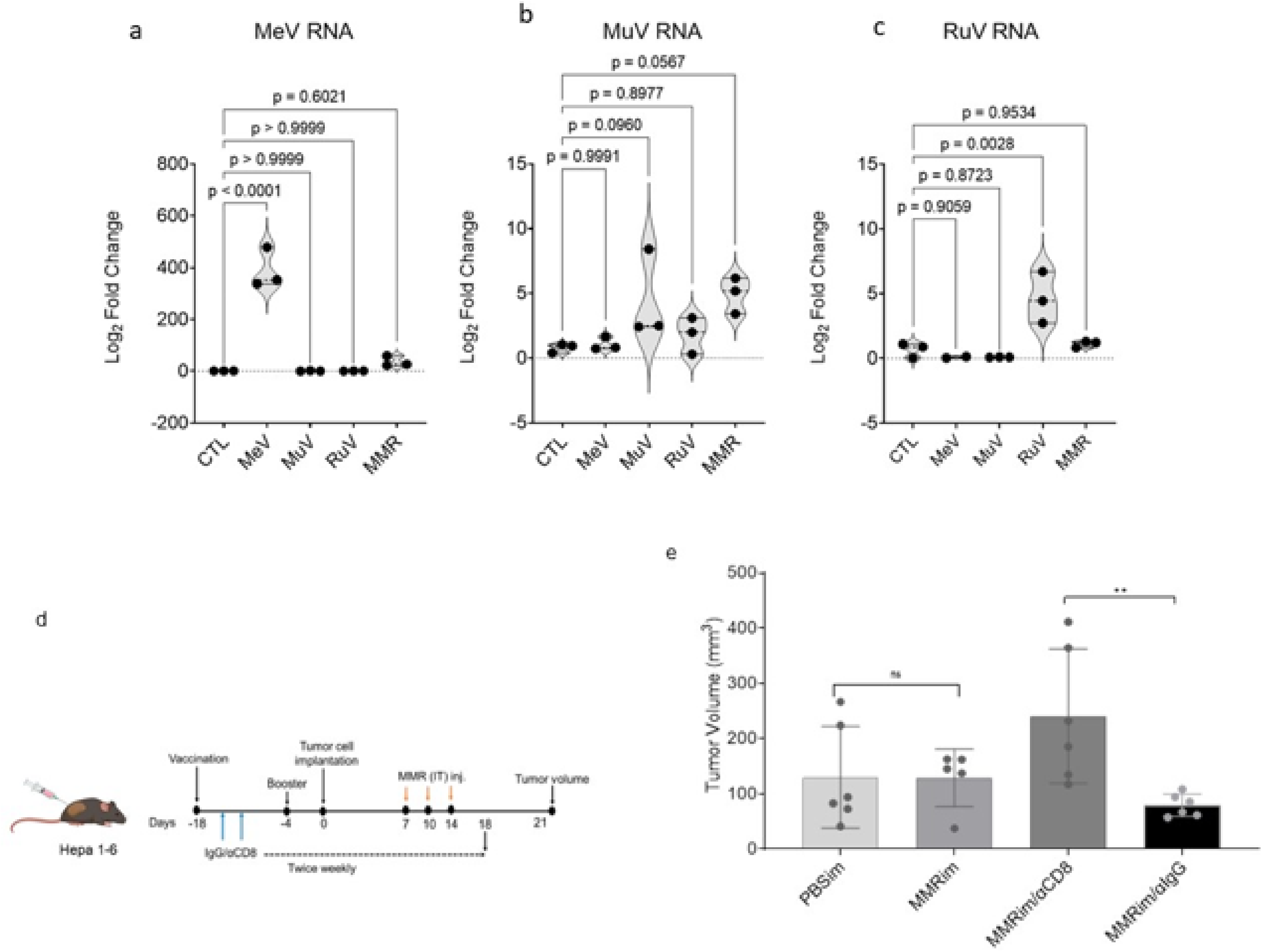

Supplementary Figure 2. Assessment of individual virus gene expression and in vivo immunization and depletion studies.

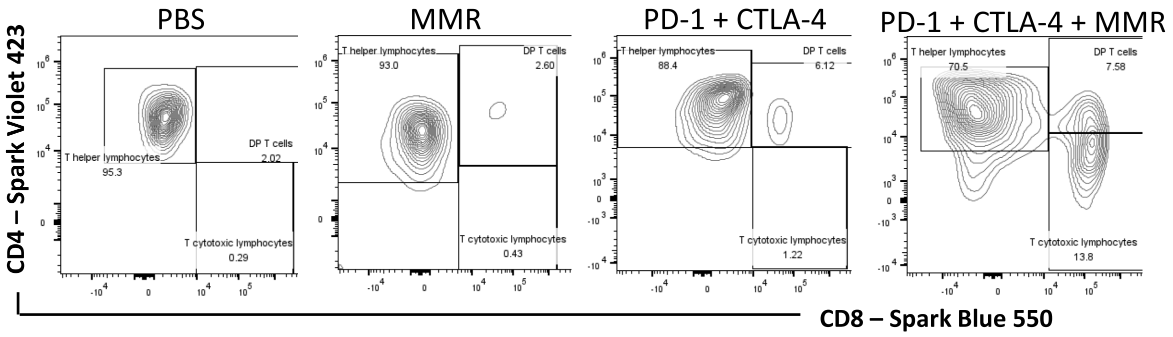

Supplementary Figure 3. Flow cytometry analysis of CD4+ and CD8+ cells in different experimental conditions.

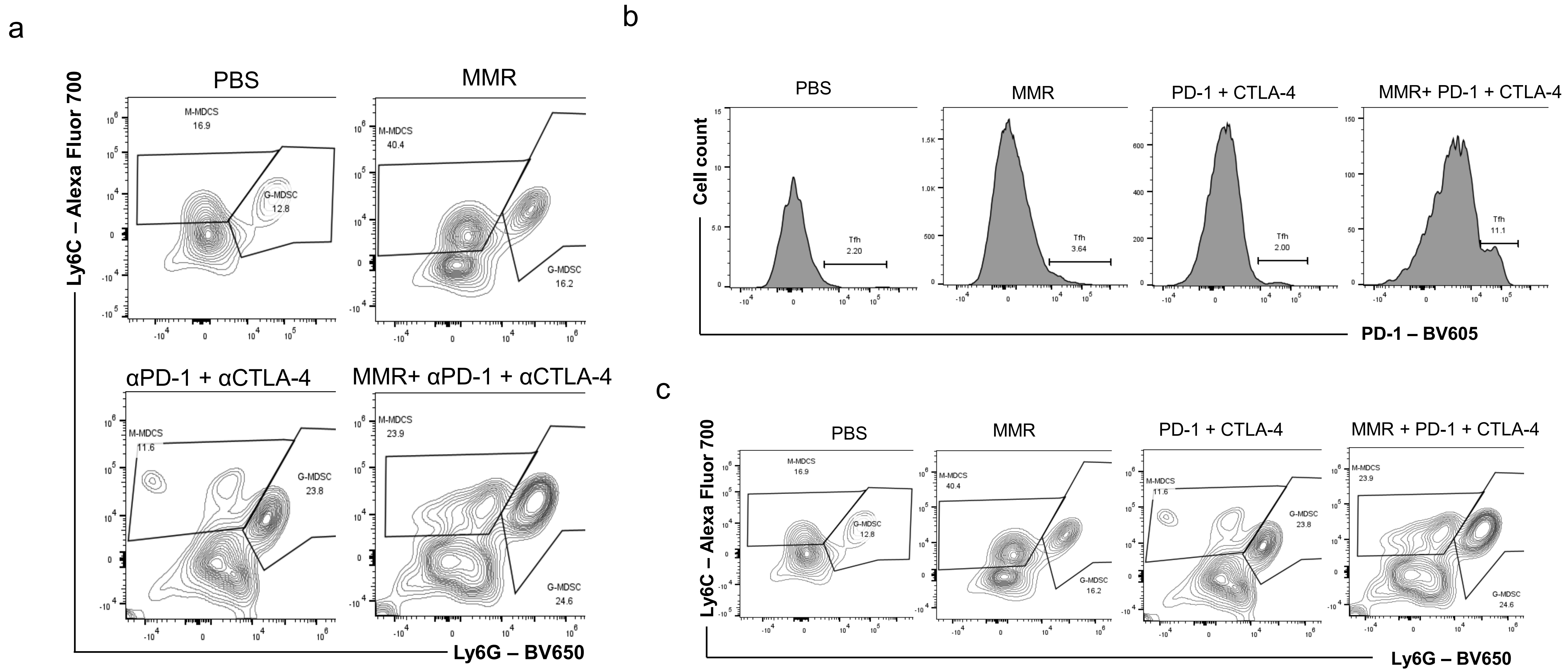

Supplementary Figure 4. Flow cytometry analysis of myeloid-derived suppressor cells in different experimental conditions.

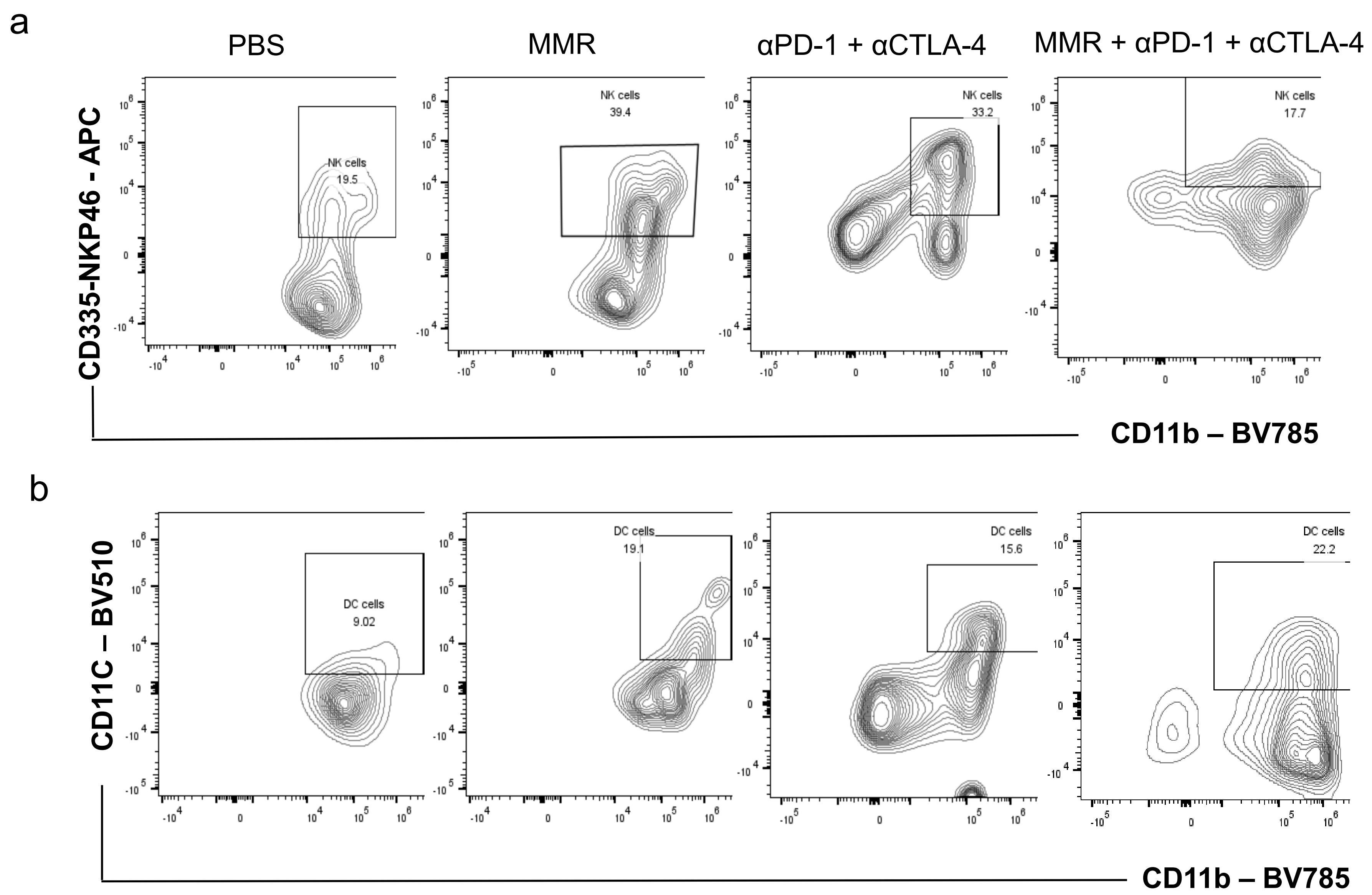

Supplementary Figure 5. Flow cytometry analysis of natural killer cells and dendritic cells across experimental conditions.

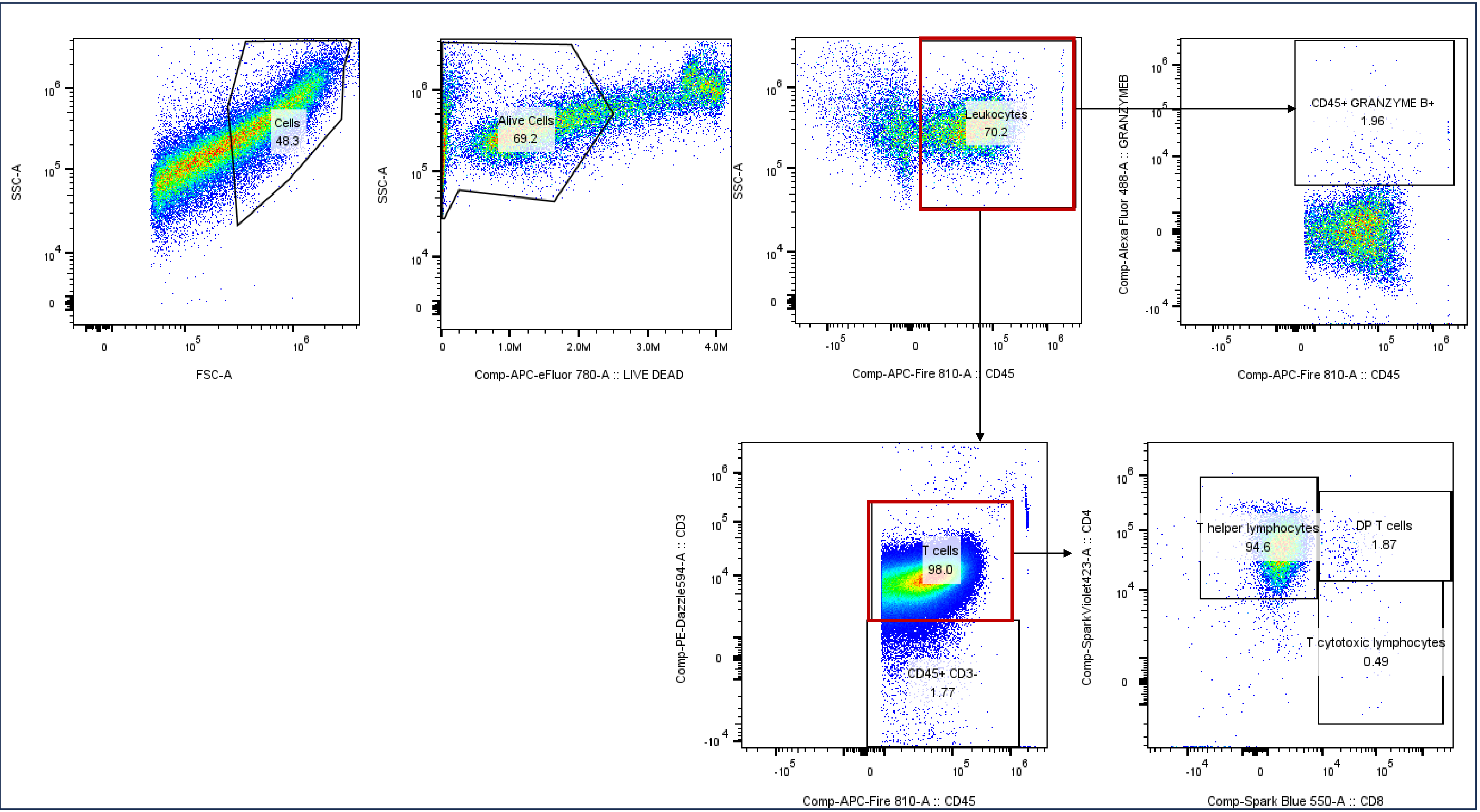

Supplementary Figure 6. Gating strategy for leukocyte, T cell, and granzyme B+ cytotoxic cell populations.

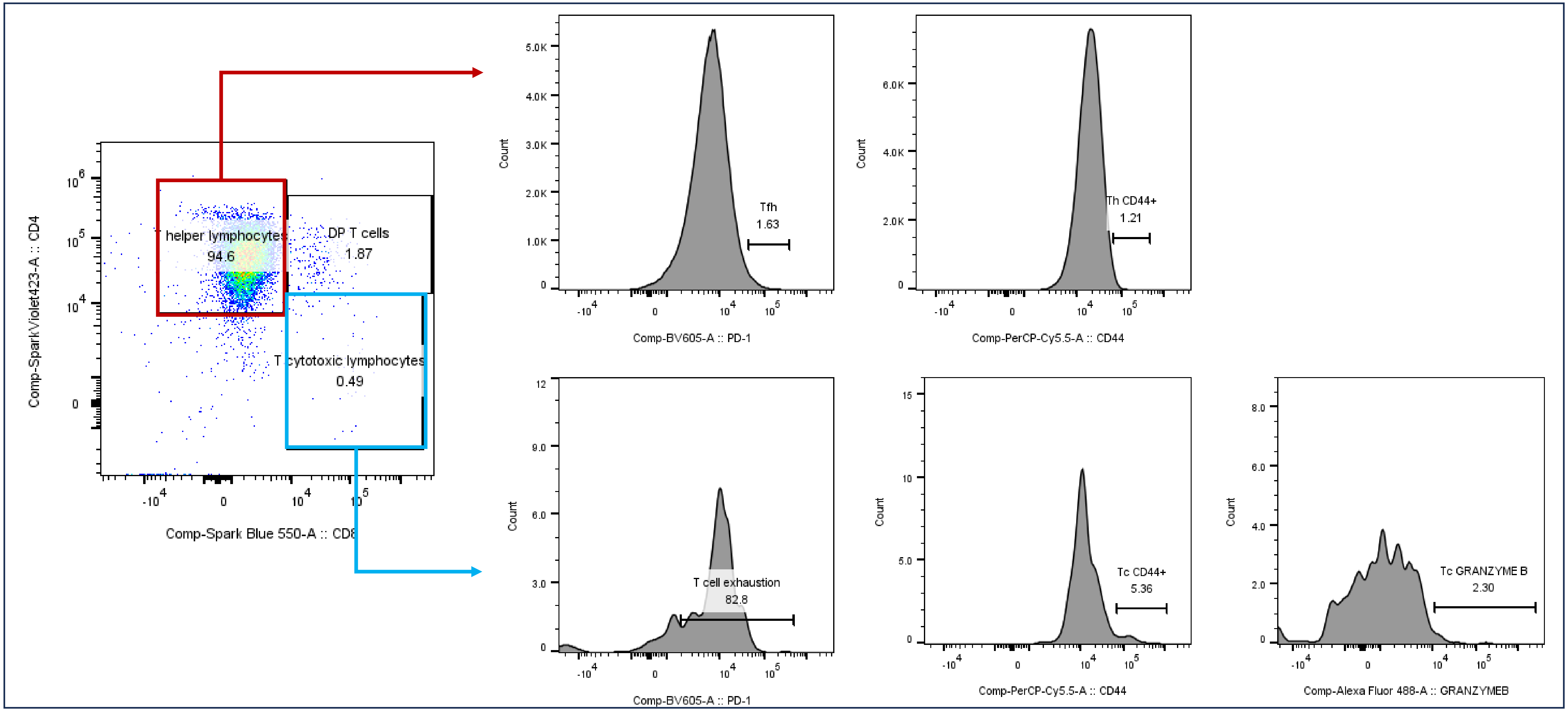

Supplementary Figure 7. Gating strategy and functional characterization of helper and cytotoxic T-cell populations.

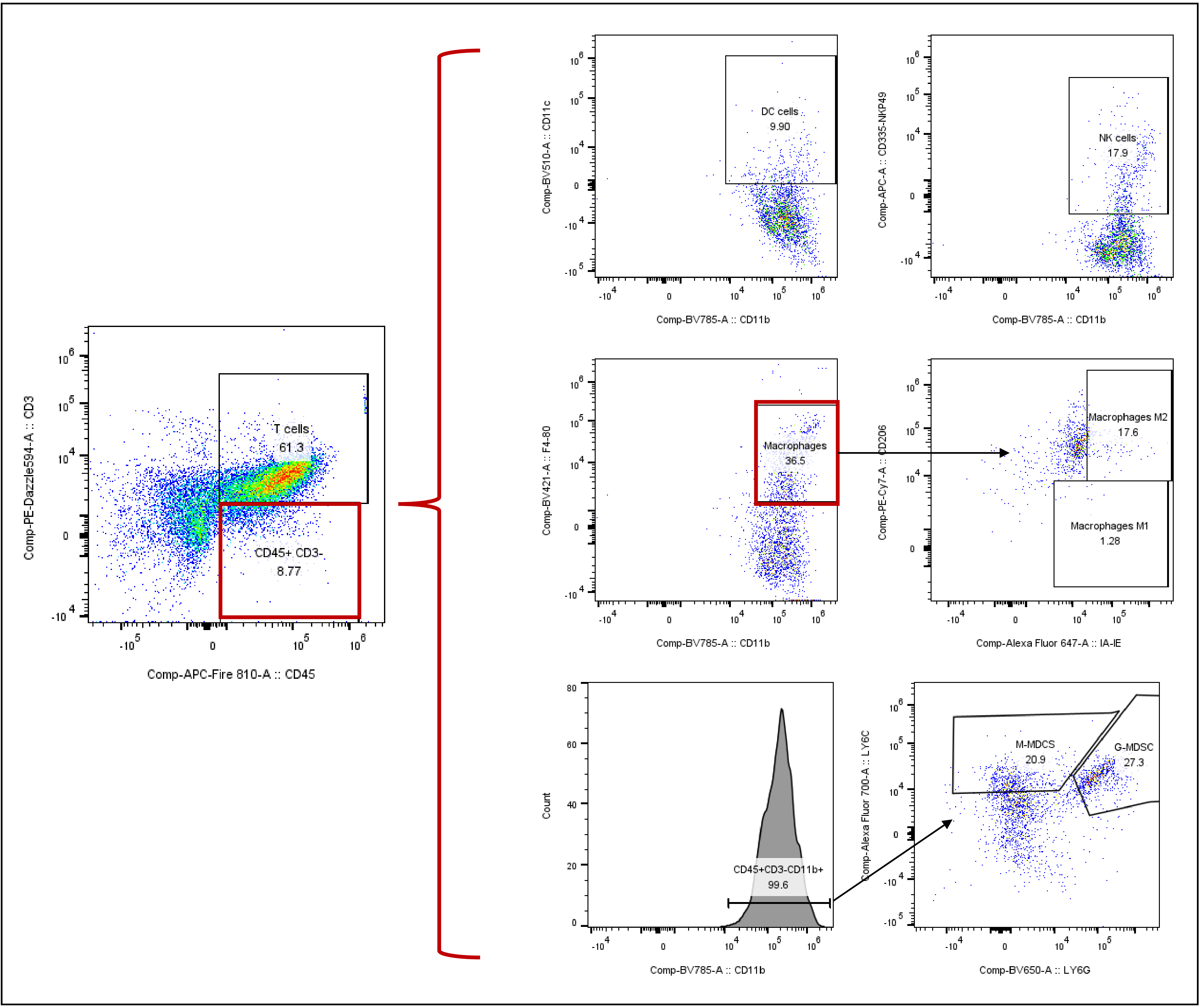

Supplementary Figure 8. Gating strategy for myeloid cell characterization: dendritic cells, natural killer cells, macrophage polarization (M1/M2), and myeloid-derived suppressor cells subset.

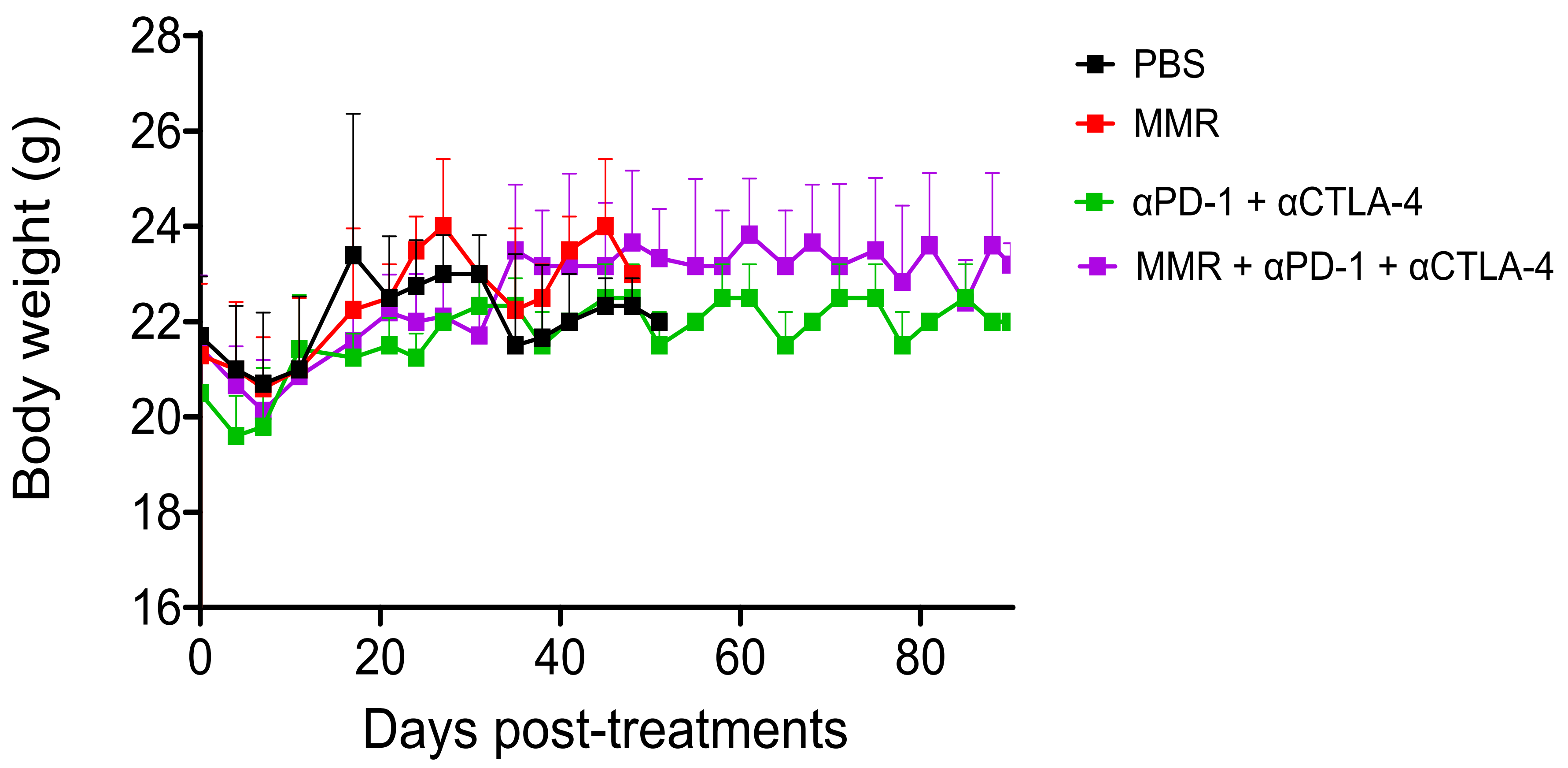

Supplementary Figure 9. Body weights across various experimental conditions in an orthotopic HCC model using R2LWT cells.

Antibody	Clone	Cat. no	Fluorochrome	Manufacturer
Live/Dead cells	-	65-0865-14	eF780	invitrogen, USA
CD45	30-F11	103174	APC-Fire 810	Biolegend, USA
CD3	17A2	100246	PE-Dazzle 594	
CD4 (L3T4)	RM4-5	100578	Spark Violet 423	
CD8a	53-6.7	100780	Spark Blue 550	
CD11b	M1/70	101243	BV785	
CD11c	N418	117353	BV510	
F4/80	BM8	123137	BV421	
CD335 (NKp46)	29A1.4	137608	APC	
CD206	C068C2	141720	PE-Cy7	
Ly6G	1A8	127641	BV650	
Ly6C	HK1.4	128024	AF700	
I-A/I-E	M5/115.5.2	107618	AF647	
CD279 (PD-1)	29F.1A12	135220	BV605	
CD44	IM7	103032	PcP-Cy5.5	
Granzyme B recombinant	QA18A28	396424	AF488	

Supplemental Table 1. List of antibodies for flow cytometry analysis. Table 1 shows the list of antibodies used for the detection and quantification of infiltrating leukocytes in tumors. Information on the product catalogue number and the provider is shown.
